# Sulfasalazine modifies metabolic profiles and enhances cisplatin chemosensitivity on cholangiocarcinoma cells in in vitro and in vivo models

**DOI:** 10.1186/s40170-021-00249-6

**Published:** 2021-03-16

**Authors:** Malinee Thanee, Sureerat Padthaisong, Manida Suksawat, Hasaya Dokduang, Jutarop Phetcharaburanin, Poramate Klanrit, Attapol Titapun, Nisana Namwat, Arporn Wangwiwatsin, Prakasit Sa-ngiamwibool, Narong Khuntikeo, Hideyuki Saya, Watcharin Loilome

**Affiliations:** 1 Cholangiocarcinoma Screening and Care Program (CASCAP), Khon Kaen University, Khon Kaen, Thailand; 2 Cholangiocarcinoma Research Institute, Khon Kaen University, Khon Kaen, Thailand; 3 Department of Pathology, Faculty of Meidicine, Khon Kaen University, Khon Kaen, 40002 Thailand; 4 Department of Biochemistry, Faculty of Meidicine, Khon Kaen University, Khon Kaen, 40002 Thailand; 5 Department of Surgery, Faculty of Medicine, Khon Kaen University, Khon Kaen, 40002 Thailand; 6 Division of Gene Regulation, Institute for Advanced Medical Research (IAMR), Keio University School of Medicine, Tokyo, 160-8582 Japan

**Keywords:** Sulfasalazine, Cholangiocarcinoma therapy, CD44v9, Metabolic signature, Chemosensitivity

## Abstract

**Background:**

Sulfasalazine (SSZ) is widely known as an xCT inhibitor suppressing CD44v9-expressed cancer stem-like cells (CSCs) being related to redox regulation. Cholangiocarcinoma (CCA) has a high recurrence rate and no effective chemotherapy. A recent report revealed high levels of CD44v9-positive cells in CCA patients. Therefore, a combination of drugs could prove a suitable strategy for CCA treatment via individual metabolic profiling.

**Methods:**

We examined the effect of xCT-targeted CD44v9-CSCs using sulfasalazine combined with cisplatin (CIS) or gemcitabine in CCA in vitro and in vivo models and did NMR-based metabolomics analysis of xenograft mice tumor tissues.

**Results:**

Our findings suggest that combined SSZ and CIS leads to a higher inhibition of cell proliferation and induction of cell death than CIS alone in both in vitro and in vivo models. Xenograft mice showed that the CD44v9-CSC marker and CK-19-CCA proliferative marker were reduced in the combination treatment. Interestingly, different metabolic signatures and significant metabolites were observed in the drug-treated group compared with the control group that revealed the cancer suppression mechanisms.

**Conclusions:**

SSZ could improve CCA therapy by sensitization to CIS through killing CD44v9-positive cells and modifying the metabolic pathways, in particular tryptophan degradation (i.e., kynurenine pathway, serotonin pathway) and nucleic acid metabolism.

**Supplementary Information:**

The online version contains supplementary material available at 10.1186/s40170-021-00249-6.

## Background

Cholangiocarcinoma (CCA) is a cancer of the bile ducts with the highest incidence occurring in northeast Thailand, where it is mainly caused by infection with the liver fluke, *Opisthorchis viverrini* (Ov) [1]. The standard treatment is surgical resection with curative intent; however, no standard for chemotherapeutic treatment has yet been established for such patients [2]. Treatment with cisplatin (CIS) plus gemcitabine (GEM) can provide a significant survival advantage of CCA patients without the addition of substantial toxicity as compared with gemcitabine alone in patients from Japan and the *UK* [2, 3]. CIS and GEM mainly act to kill proliferating cancer cells, but not cancer stem-like cells, with an interruption of the DNA or RNA synthesis [4–6]. The pathogenesis of CCA depends on the causes of the disease, especially the presence or absence of Ov infection. The different molecular mechanisms of Ov- and non-Ov-infection-associated CCA showed that the major factor promoting Ov-associated CCA development is inflammation, whereas for the non-Ov-associated CCA, this is mainly caused by a growth factor [7]. In addition, the aforementioned study showed that Ov-associated CCA cell lines are more resistant to chemotherapeutic drugs such as cisplatin than non-Ov-associated CCA cell lines [8, 9]. Therefore, Ov-associated CCA is more aggressive and more resistant to chemotherapeutic drugs than non-Ov-associated CCA.

Cluster of Differentiation 44 (CD44) is used as a cell surface marker in order to identify cancer stem-like cells (CSCs) in many cancer types [10–13]. Importantly, a variant of CD44 could stabilize xCT (a cystine-glutamate transporter) linked to the ROS defense system via cystine uptake-mediated glutathione synthesis [14]. A previous report indicates that the redox status regulation of CCA cells depends on the expression of CD44 variant 9 (CD44v9) that is associated with the xCT function contributed to redox control and is a link to the poor prognosis of patients [15]. Moreover, CD44 has a co-interaction function with Pyruvate Kinase M2, regulating cell proliferation via modifying glucose metabolism [16, 17]. The reduction of CD44 could modify cellular metabolism [18]. Taken together, CD44 plays a crucial role in cancer metabolism, including the alteration of amino acids, glucose, and redox metabolism.

Sulfasalazine (SSZ) is a well-characterized specific inhibitor of xCT-mediated cystine transport and has been shown to selectively suppress the proliferation of CD44v9-positive cancer cells [19]. It is a drug usually administered for ulcerative colitis or rheumatoid arthritis [20, 21]. SSZ could inhibit CD44v9-positive CCA cell proliferation and stimulate CCA cell death via a reduction of glutathione (GSH), consequently increasing intracellular ROS levels and inducing the phosphorylation of p38 mitogen-activated protein kinase, an indicator of intracellular ROS levels [15]. Currently, phase I clinical trials and some cancer patients treated with sulfasalazine have shown a reduction in CD44v9-positive cells and the intra-tumoral glutathione level [22]. Sulfasalazine can be used safely in cisplatin treatment combined with pemetrexed to prolong progression-free survival [23**]**.

Metabolomics is an omics technology in system biology used to detect phenotypic changes and reflect the state of the cell using nuclear magnetic resonance (NMR) spectroscopy through the quantitative measurement of small molecular weight metabolites including sugars, nucleotides, nucleic acid, and lipids [24, 25]. Currently, the medical advantages of metabolomics are to discover potential biomarkers for the early detection and diagnosis in colon [26] and ovarian cancer [27]. Additionally, it can be used to find predictive markers for the evaluation of a patient’s response to drugs [28]. It can also be used to provide evidence supporting a better understanding of molecular mechanisms [29]. In addition, it has been reported that most drugs induce many metabolic changes, reflecting their effect on multiple interconnected metabolic pathways and networks [30]. Furthermore, pharmacometabolomics research for drug response phenotyping, as influenced by the environment, genetics, and gut microbiome, contribute to pharmacology, clinical pharmacology, drug discovery and development, clinical trials, and precision medicine [30, 31]. Therefore, metabolic signatures provide new insights into the mechanisms of drug action and can be used as biomarkers for drug response phenotypes leading to increased success in choosing a drug treatment.

Taken together, SSZ-targeted to CD44v9-positive cells might contribute to sensitize these cells to anti-cancer treatment via regulating their redox status. Hence, the effect of SSZ on sensitizing cells to chemotherapeutic drugs in CCA, both in vitro and in vivo, and their metabolic signatures of the different drug treatments was investigated.

## Methods

### Cell culture and reagents

The human cholangiocarcinoma cell lines KKU-213 and KKU-100 were established from CCA patients of Srinagarind Hospital, Khon Kaen University, and purchased from the Japanese Collection of Research Bioresources (JCRB) Cell Bank, Osaka, Japan. KKU-213 is a mixed (papillary and non-papillary) cholangiocarcinoma which was established from a 58-year-old male patient, whereas KKU-100 is a poorly differentiated cholangiocarcinoma established from a 65-year-old female patient. All cell lines were grown in DMEM medium (Gibco Life Technology, *Carlsbad,* CA, USA), supplemented with NaHCO_3_, 100 units/ml penicillin, 100 mg/ml streptomycin, and 10% fetal bovine serum at 37 °C containing 5% CO_2_ in a humidified incubator. Three reagents were used: SSZ, an inhibitor of xCT (Sigma-Aldrich, MO, USA), cisplatin purchased from Boryung Pharmaceutical Co., Ltd. (Gyeonggi-do, South Korea), and gemcitabine purchased from the Eli Lilly Corporation (Indianapolis, IN, USA).

### Immunohistochemical staining

Liver tissues were fixed in 10% buffered formaldehyde, embedded in paraffin blocks, and then sectioned at a thickness of 4 μm. Sections were deparaffinized in xylene and rehydrated in an ethanol series. Immunohistochemical staining was performed for CD44 variants 9 (CD44v9), and Ki-67 (proliferative marker) according to standard methods as previously described [32, 33]. Anti-CD44v9 was purchased from Cosmo Bio (1:50 dilution, Cosmo Bio, Tokyo, Japan) and Ki-67 was purchased from Abcam (1:300 dilution, Abcam, *Cambridge, UK*). The sections were observed under a light microscope at ×200 and ×400 magnifications (Axioscope A1, Carl Zeiss, Jena, Germany). The scoring system of IHC was performed as previously described [34].

### Immunofluorescence analysis

The tissue sections were processed for immunohistochemical staining and retrieved by heating in 0.01 M sodium citrate containing 0.05% Tween 20 (pH 6.0) for 10 min at 110 °C. The samples were then exposed to 3% bovine serum albumin before being incubated at 4°C overnight with primary antibodies: CD44v9 (1:50 dilution, Cosmo Bio, Tokyo, Japan) and CK-19 (1:300 dilution, Abcam, *Cambridge, UK*). After washing with PBS, the samples were incubated with Alexa Fluor 488- or 555-conjugated secondary antibodies (Invitrogen, Waltham, MA, USA) and mounted in Hoechst 33342 (Invitrogen, Waltham, MA, USA). The fluorescence signals were detected under a Zeiss LSM800 confocal laser scanning microscope (Carl Zeiss, Oberkochen, Germany).

### Tunel assay

The tumor tissue sections were deparaffinized and rehydrated using xylene, and 100%, 90%, 80%, 70% alcohol twice each for 5 min, then incubated with proteinase K (20 ug/ml in 10 mM Tris-HCl pH 7.4) for 30 min in a 37 °C incubator. The samples were then exposed to hydrogen peroxide before being incubated at 37°C for 60 min with TUNEL reaction mix (Enzyme solution 50 uL + nucleotide mixture solution 450 uL). After washing with PBS, the samples were incubated with convertor-POD at 37 °C for 30 min. The signals were detected using the DAB system and observed under a light microscope (Axioscope A1, Carl Zeiss, Jena, Germany).

### Flow cytometry analysis for the apoptosis assay

KKU-213 cells were seeded at 5 × 10^5^ cells/well. After the cells had adhered, they were treated with the drug for 48 h. Both dead and living cells were collected then washed with 1×PBS. The cells were incubated with annexin V and propidium iodide (Sigma-Aldrich, MO, USA) for 15 min. The signal of positive cells was detected with Flow cytometry analysis using FACSCanto II (BD Bioscience, San Jose, CA, USA).

### Cell proliferation and cell cytotoxicity

The number of viable cells was evaluated with a Cell Titer-Glo luminescence cell viability kit (Promega, Madison, WI, USA). Briefly, CCA cells (2x10^3^ cells per well) were plated into 96-black well plates for 24 h. Cells were then treated with SSZ (0, 200, 400, 600, 800, 1000 μM) and CIS (0, 10, 20, 40, 60, 80, 100 μM) for 48 h, and GEM (0, 10, 20, 40, 60, 80, 100 μM) for 72 h. In addition, 300 μM of SSZ was used in combination with CIS or GEM. The luminescence signal was detected on a SpectraMaxL microplate reader. The experiments were done in triplicate.

### Animal model

The Animal Ethics Committee of Khon Kaen University (AEKKU 6/2560) approved the study protocol. Female nude mice (4- to 6-week-old purchased from Nomura Siam International CO., Ltd., Japan) were used to establish subcutaneous xenograft mice. They were injected subcutaneously with 2 × 10^6^ KKU-213 CCA cells on both flank sides. This cell line previously demonstrated highly expressed CD44v9 protein. A day after tumor injection, the mice were orally administered either a vehicle or sulfasalazine (250 mg/kg body weight) daily for 15 days. In the case of cisplatin treatment, the mice were injected intravascularly with cisplatin (2 mg/kg body weight) twice a week. The body weight of each animal was observed and tumor masses were removed and weighed 22 days after inoculation.

### ^1^H NMR analysis of tissue extraction

To extract tumor tissues for detection of metabolomics, we weighted 100 mg of the tumor tissue and washed this with 1×PBS pH 7.4. The samples were then extracted with methanol and chloroform, the supernatant was separated into a polar phase and a lipophilic phase after centrifugation at 1000 g for 15 min. The solvents were removed using a speed vacuum concentrator (Labconco, Kansas City, MO, USA). The tissues were re-suspended with 560 μl of 100 mM sodium phosphate buffer, pH 7.4 in D_2_O containing 0.1 mM 3-trimethysilypropionic acid (TSP) (Cambridge Isotype Laboratories, Tewksbury, MA, USA) as a chemical shift reference (*δ* = 0 p.p.m.) and optionally 0.2% NaN_3_. Next, the extracted tissue samples were transferred to a NMR tube and after being vortex centrifuged at 12,000 g for 5 min. Proton NMR spectra were acquired using a 400 MHz NMR spectrometer (Bruker, Ettlingen, Germany). All samples were detected using a standard 1-dimensional pulse sequence (recycle delay-90°-t1-90°-tm-°-acquisition) with t1 to 3 ms, tm to 10 ms, abd 90° pulse to 10 μs in 32 scans.

### Statistical analysis

MATLAB (R2015a) was used for multivariate analysis, both unsupervised and supervised multivariate statistical methods, and in-house developed scripts were employed. The unsupervised analysis involved principal component analysis (PCA) which generates a model showing the intrinsic similarities or differences without prior class information and reduces the complexity and intricacy of the data. Orthogonal projection to latent structures-discriminant analysis (OPLS-DA) was the supervised multivariate statistical method. This analyses the statistical models of class membership data which is then used to optimize the separation between the different classes. The fitness and predictability of the OPLS-DA models were determined by *R*^2^ and *Q*^2^ values. The assignments of discriminatory metabolites were confirmed using ChenomxNMR Suite software analysis and statistical total correlation spectroscopy (STOCSY) on 1-dimensional NMR spectra. SPSS software version 17.0 (IBM Corporation, Armonk, NY, USA) was used for statistical analysis. The differences among each group of samples were analyzed using a *t*-test. The data were expressed as a graph of mean ± S.D. using Graph Pad prism 5. All analyses were two-tailed and *p*-values < 0.05 were considered statistically significant.

## Results

### SSZ helps CIS drugs to kill CCA effectively via activation of cell apoptosis not sensitized to GEM treatment

**Fig. 1:**
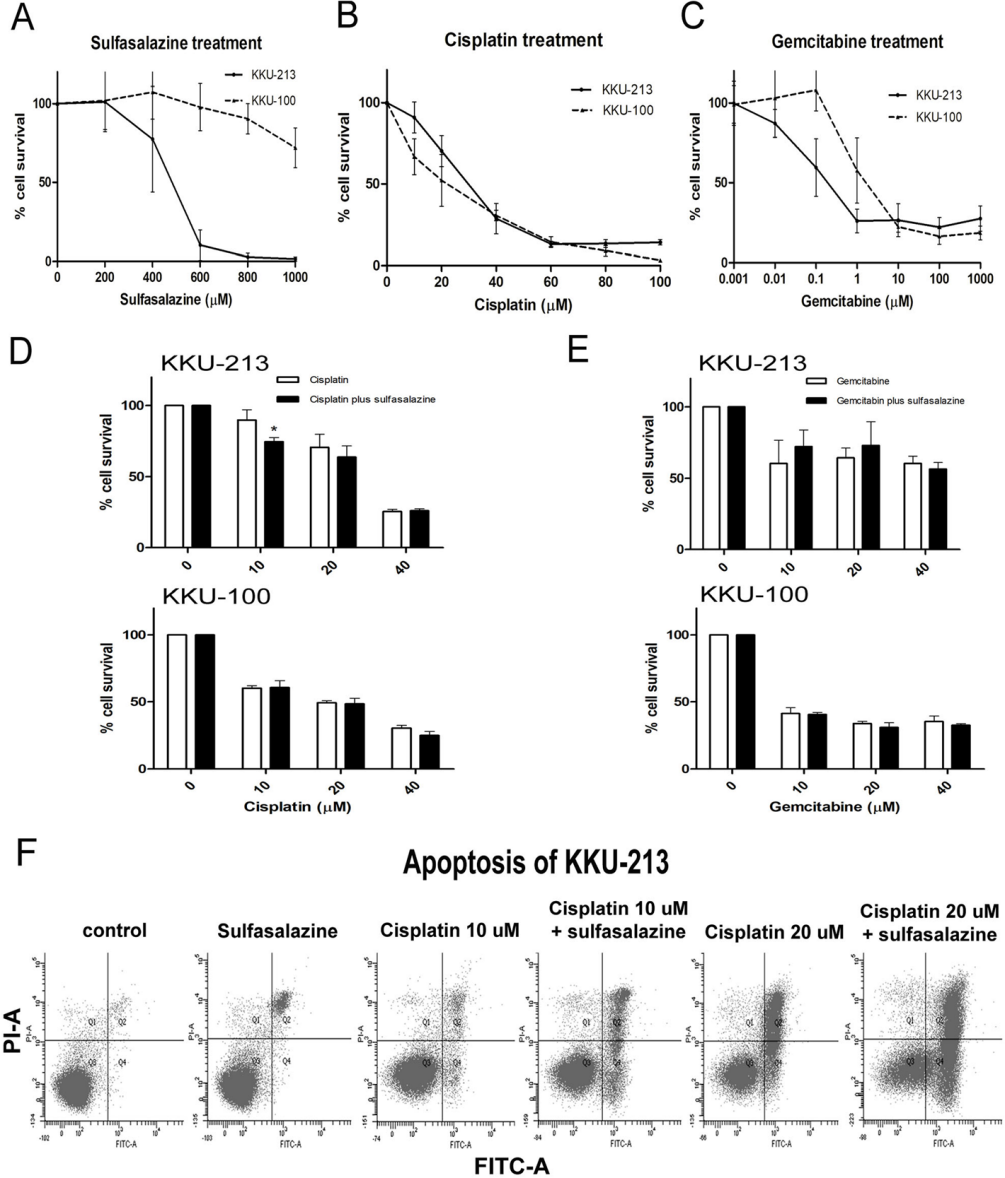
Sulfasalazine, xCT inhibitor, sensitizes to cisplatin chemotherapeutic drugs to kill CCA cells. **a** Percentage of CCA cell survival after cells were treated with various concentrations of sulfasalazine, **b** cisplatin, and **c** gemcitabine in CCA cell lines KKU-213 and KKU-100. This was determined by a Cell Titer-Glo luminescence cell viability kit. **d** CCA cells treated with a combination of cisplatin or **e** gemcitabine and sulfasalazine: the luminescence signal of living cells was detected and calculated to be the cell survival percentage as shown in the bar graph. **f** A cell population stained with PI and annexin-5 after drug treatment detected by flow cytometry in KKU-213 cell line. Data are the mean ± standard deviation of independent, triplicate experiments

We examined the effect of SSZ, which is targeted on the CD44v9-xCT system, in sensitizing cells to the available chemotherapeutic drug treatment with CIS and GEM. A combination of SSZ with CIS significantly enhanced the cytotoxicity of CIS at 10 μM and 20 μM in KKU-213, but this was not found in KKU-100. On the other hand, a combination of SSZ with GEM did not enhance the cytotoxic effect in any of the CCA cell lines (Fig. 1d and e).

Based on the cytotoxicity of CIS combined with SSZ, we further investigated the effect of SSZ combined with CIS on apoptosis using flow cytometry with KKU-213 cells. The number of apoptotic cells with CIS at 10 μM and 20 μM in combination with SSZ was significantly higher than CIS alone (Fig. 1f and Additional file 1: Figure S1).

### Tumor growth was suppressed with CIS in combination with SSZ in the in vivo model

**Fig. 2:**
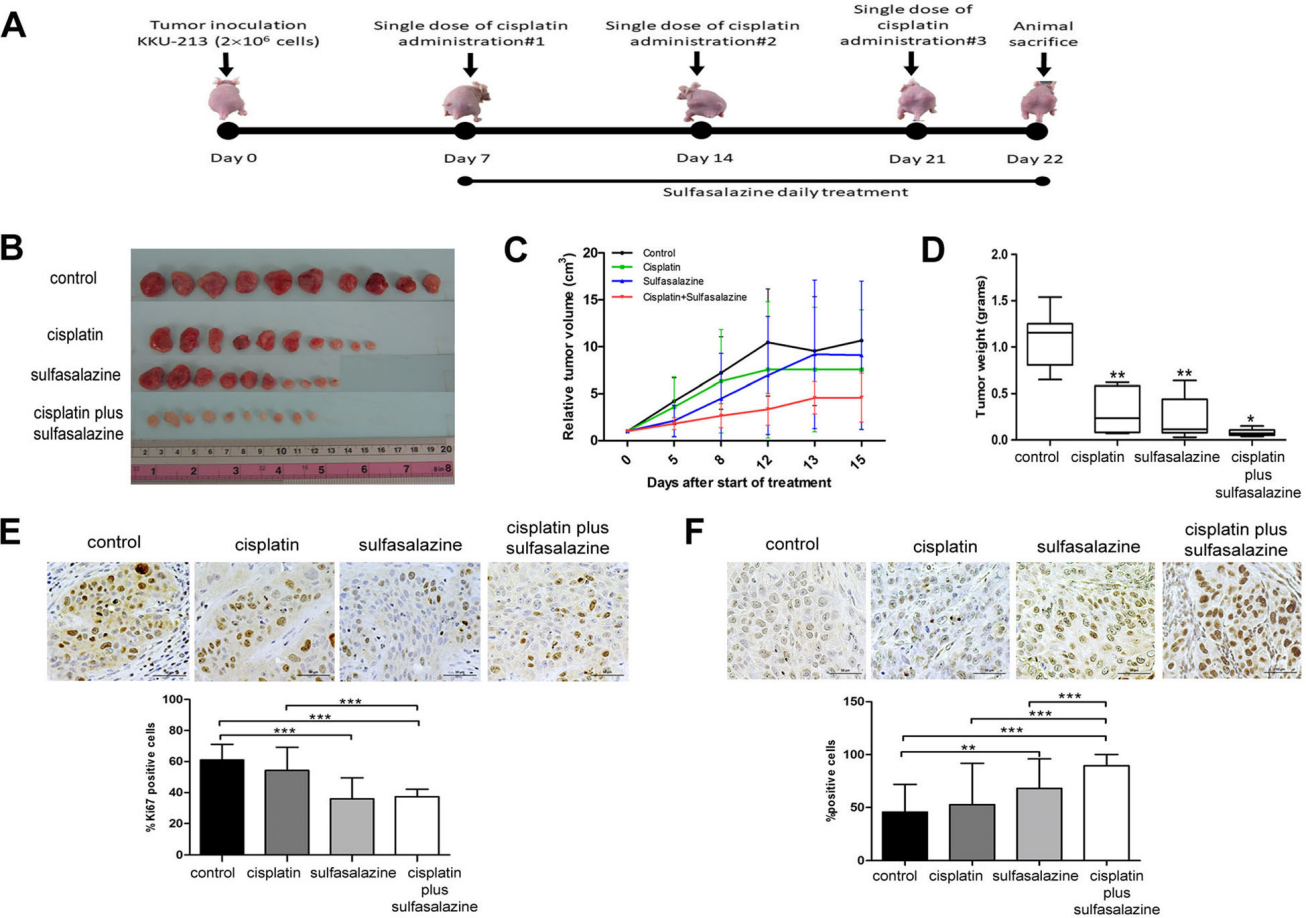
SSZ inhibits cell growth and activates cell death in the in vivo model. **a** Time line of nude mice treatment with cisplatin in the absence/present of sulfasalazine. **b** The tumor mass after treatment. **c** The tumor volume expressed as the mean ± standard deviation of 10 tumor masses in 5 nude mice in each group. **d** The tumor weight expressed as the mean ± standard deviation of 10 tumor masses in 5 nude mice in each group. **e** The proliferation index indicated by Ki67 staining-positive cells, and the percentage represented as the mean±standard deviation of 5 nude mice in each group. **f** Apoptosis was measured by TUNEL assay and the percentage is presented as the mean±standard deviation of 5 nude mice in each group

We selected highly CD44v9-expressed KKU-213 cells to subcutaneously inject into nude mice. The body weight of the drug-treated nude mice was not significantly different from the control mice (Additional file 1: Figure S2). The nude mice treated with CIS with the absence/presence of SSZ showed a higher inhibitory effect on tumor volume and tumor weight with a combination of CIS and SSZ compared with CIS or SSZ alone (Fig. 2b–d). The proliferation index based on Ki67 staining revealed that the percentage of Ki67-positive cells in the tumor tissue of either CIS or SSZ was reduced when compared with the untreated group (Fig. 2e). The percentage of Ki67-positive cells in the tumor tissue treated with a combination of CIS and SSZ was significantly lower than either the CIS or SSZ group (Fig. 2e). The measurement of apoptotic cells by TUNEL assay showed that the percentage of apoptotic cells in the combination treatment was significantly higher than either the CIS or SSZ treated group (Fig. 2f). Moreover, the percentage of apoptotic cells in the tumor tissue of CIS or SSZ, or the combination treatment, was significantly higher than in the untreated group (Fig. 2f).

### SSZ reduces CD44v9 expressing cells resulting in cell growth inhibition in the in vivo model

**Fig. 3:**
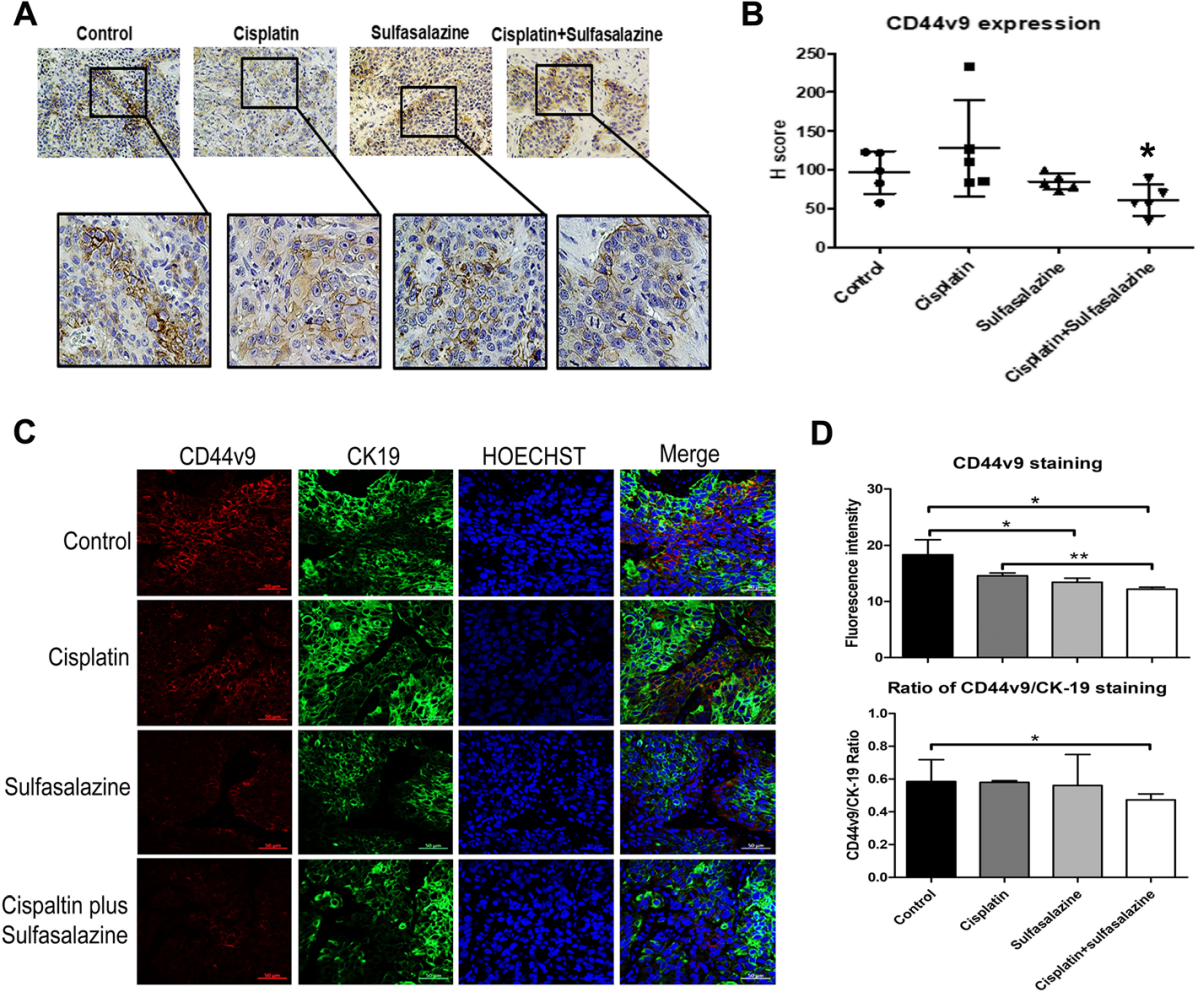
Cancer stem cell makers are reduced after treatment in the in vivo model. **a** Immunohistochemical staining of CD44v9. **b** The graph indicates the grading score of CD44v9 expression as the mean±standard deviation of 5 nude mice in each group.**c** Co-localization of CD44v9 and CK-19 with immunofluorescence staining. **d** The bar graphs indicate the fluorescence intensity of CD44v9 staining and the ratio of CD44v9 and CK-19

The expression of the cancer stem cell marker was reduced after treatment in the in vivo model. Previously, we demonstrated that 95% of CCA patients express CD44v9 while 43% of these patients have high expression [15]. Immunohistochemical staining of CD44v9 showed that the level decreased with a combination of CIS and SSZ when compared with CIS alone (Fig. 3a and b). Interestingly, co-localization of CD44v9 and the proliferative marker of CCA, CK-19, with immunofluorescence staining indicated that both proteins in the combination group were less expressed than with CIS or SSZ alone (Fig. 3c). The ratio of CD44v9 to CK-19 in a combination of CIS and SSZ was significantly less than CIS alone (Fig. 3d).

### Treatment with CIS or SSZ or a combination of the two drives the alteration of the metabolic profile and is associated with cell growth inhibition in the in vivo model

**Fig. 4:**
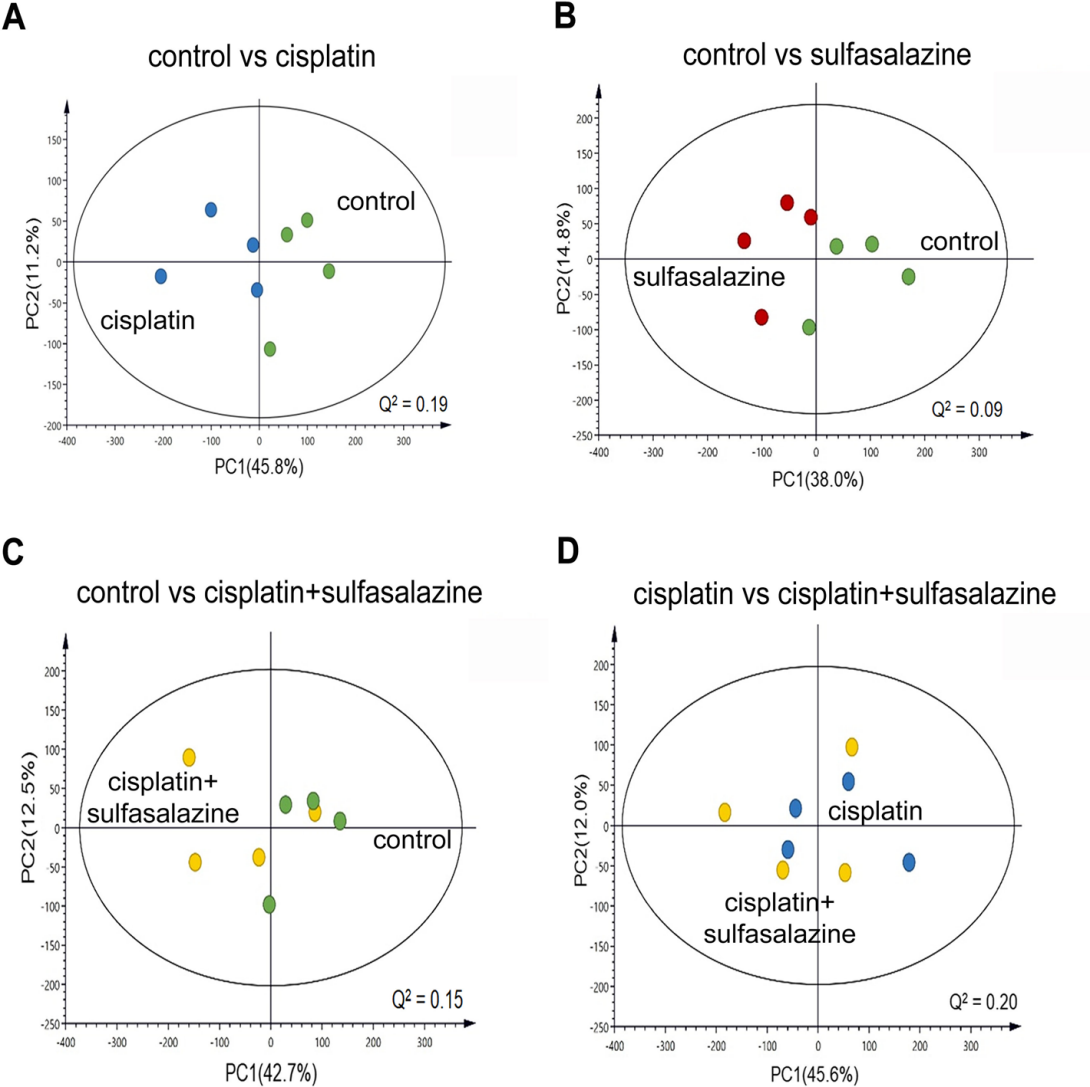
Multivariate analysis using Principle Component Analysis (PCA) plot between the control and treatment groups. **a** Cisplatin and **b** sulfasalazine treatment could be distinguished from the untreated group in the principal component score chart. This indicates that the metabolic profiling of both was different from that of the untreated control, t [1] and t [2] are scores on PC1 and PC2, respectively. **c** The components of cisplatin combined with sulfasalazine treatment did not differ when compared with the untreated group and **d** cisplatin alone

The chemical shift of representative metabolites in tumor tissues in response to drugs follow different patterns (Additional file: Figure S3); data were consequently analyzed using principal component analysis (PCA), an unsupervised pattern recognition algorithm. Our results revealed that a score plot between each group can be distinguished by the first two principal components (PC1 and PC2), suggestive of a metabolic profile change after treatment (Fig. 4).

**Fig. 5:**
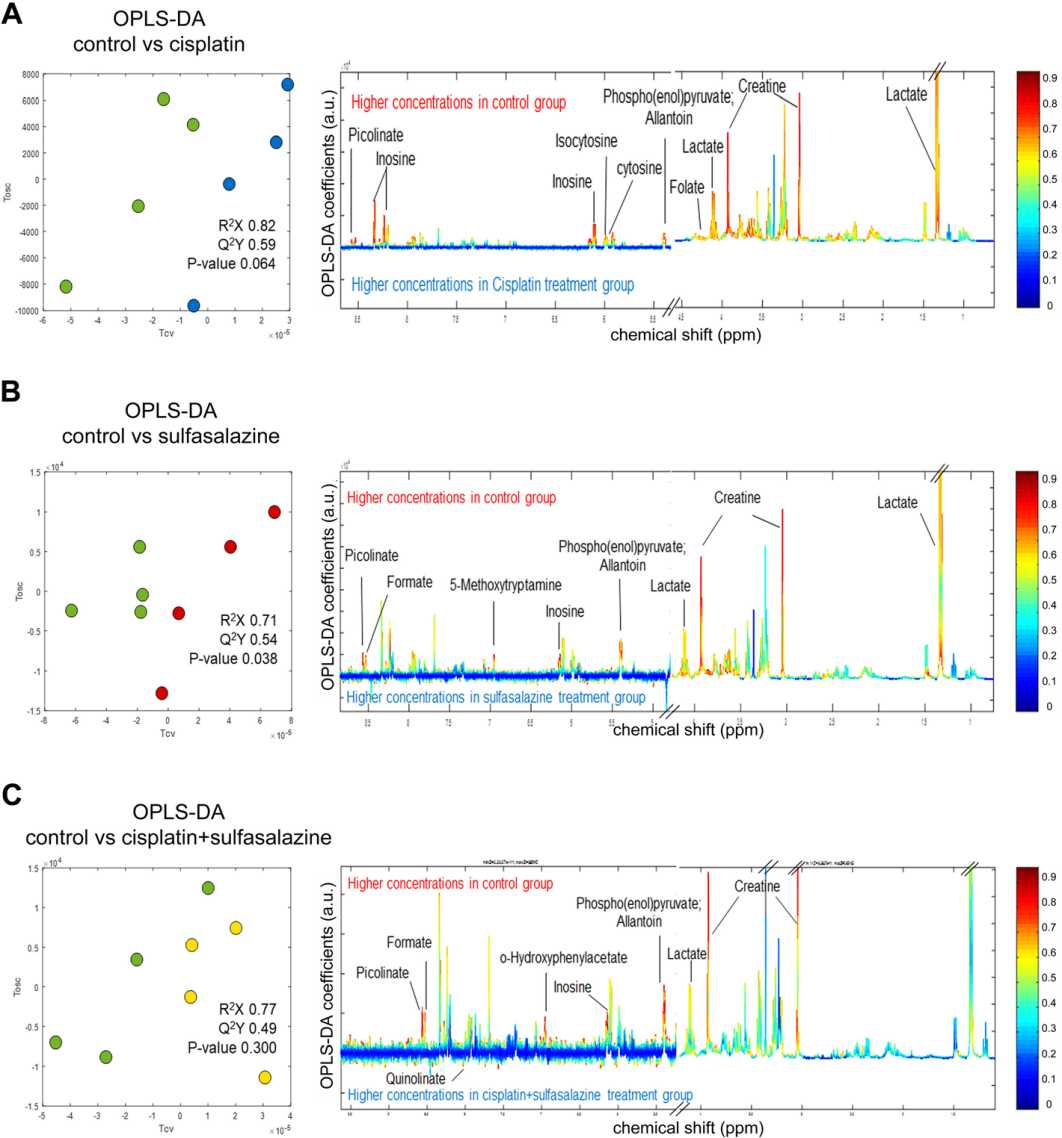
OPLS-DA analysis distinguished metabolites between control and treatment with cisplatin, or sulfasalazine, or a combination of both. **a** OPLS-DA analysis shows that the cross-validation plot and coefficient loading plots derived from 1 H NMR spectra of control and treatment samples were altered in the control and cisplatin models, **b** control and sulfasalazine, and **c** control and cisplatin plus sulfasalazine. The upper section (above 0) of the loadings plot characterizes metabolites higher for the control, whereas the lower section (below 0) represents metabolites that are higher for the treatment groups. The color of each peak relates to the correlation value of the metabolites in the discrimination model

In addition, a supervised pattern recognition algorithm was applied using orthogonal partial least squares discriminant analysis (OPLS-DA). The OPLS-DA model, which is the statistical modeling method generated by rotating principal component projection to filter inappropriate data, can provides insights to distinct two individual groups (Fig. 5). Furthermore, differentially expressed metabolites between the control and CIS or the control and SSZ, as well as the control and CIS plus SSZ were distinguished by coefficient loading plots as shown in Fig. 5. The coefficient of determination or *R*-squared (*R*^2^) shows a *statistical* measure of our regression model in Table 1. Our findings indicate that the common metabolites in response to all drugs were creatine, allantoin, inosine, picolinate, and phosphocreatine, while other metabolites that were different in response to CIS or SSZ or a combination of the two were 3-hydroxykynurenine, alanine, glycerate, lactate, NAD^+^, phosphor (enol)pyruvate, uracil, cis-aconitate, cytosine, isocytosine, orotate, 5-hydroxytryptophan, anserine, p-hydroxyphenylpyruvate, tryptophan, and *N*-acetylhistamine. All metabolites were higher in the untreated control samples, except for quinolinate and indole-3-pyruvate, which were higher in the SSZ treated group and the CIS plus SSZ treated group; these were not seen in CIS alone.

**Table 1:** Metabolite profiling of CCA tissues

Metabolites	Chemical shift	***R***^**2**^ of OPLS-DA			***R***^**2**^ of OPLS regression
		Control vs cisplatin	Control vs SSZ	Control vs combined	Tumor size of control vs combined
3-Hydroxykynurenine	3.71(d); 4.16(t); 6.71(t); 7.05(d); 7.45(d)		0.8822		
Alanine	1.48(d); 3.79(q)				
Glycerate	3.72(dd); 3.83(dd)		0.8940		
Creatine	3.04(s); 3.93(s); 4.09(dd)	0.9217	0.9039	0.8967	0.8967
Lactate	1.33(d); 4.11(q)	0.8523	0.7985		
NAD^+^	4.761(t); 6.094(d); 8.239(s); 8.335(s)	0.8593			
Phospho(enol)pyruvate	5.19(t); 5.37(t)	0.9175	0.8901	0.857	
Allantoin	5.4(s)	0.8547	0.8083	0.8688	0.877
Uracil	5.81(d); 7.54(d)			− 0.6106	
Unknown-1				0.8738	0.9039
cis-Aconitate	3.17(s); 5.92(s)	0.8518			
Cytosine	5.98(d); 7.51(d)	0.8090			
Isocytosine	5.99(d); 7.62(d)	0.8894			
Inosine	3.85(dd); 3.92(dd); 4.28(q); 4.44(t); 6.1(d); 8.24(s); 8.34(s)	0.9123	0.9887	0.9754	0.9603
Orotate	6.2(s)				− 0.8591
5-Hydroxytryptophan	3.23(dd); 3.41 (dd); 4.02(dd); 6.87(d); 6.88(d); 7.14(s); 7.28(s); 7.41(d)				0.9361
Anserine	2.67(m); 3.01(dd); 3.21(m);3.21(dd);3.72(s);4.48; 6.97(s); 7.95(s)			0.8258	
p-Hydroxyphenylpyruvate	3.92(s); 6.63(d); 6.99(d); 7.73(d); 9.43(s)			0.7812	
Tryptophan	3.31(dd); 3.49(dd); 4.06(dd); 7.21(t); 7.29(t); 7.33(s); 7.55(d); 7.74(d)			− 0.8846	
Quinolinate	7.45(q); 7.9(d); 8.02(d)			− 0.7986	
N-Acetylhistamine	2.84(t); 3.45(m); 7.03(s); 7.95(s)	0.8164			
Picolinate	7.54(t); 7.92(d); 7.96(t); 8.57(s)	0.9170	0.8502	0.8232	0.8232
Phosphocreatine	3.05(s); 3.95(s)	0.8443	0.9367	0.8623	0.8835

*s* singlet, *d* doublet, *t* triplet, *q* quartet, *dd* doublet of doublet, *m* multiplet

The treatment group of KKU-213 subcutaneously injected nude mice was divided into 2 subgroups, including response and non-response, depending on the drug response using tumor weight determination after treatment. In response to CIS, the general metabolites found were NAD^+^, cis-aconitate, cytosine, isocytosine, and N-acetylhistamine. 3-Hydroxykynurenine was found only in the SSZ response group. However, the shared metabolite that was found in the CIS and SSZ response groups was lactate. Importantly, uracil, anserine, p-hydroxyphenyl pyruvate, and tryptophan were seen only in the combination treatment. Interestingly, the different metabolites in the control and combination groups based on tumor weight, which distinguished them from other pairs, were orotate and 5-hydroxytryptophan. Unfortunately, although we could detect the common metabolite that was associated with tumor weight and differed from the control metabolites, we could not identify it as it still has the name unknown-1 (Table 1).

By calculating the spectral peaks of the corresponding metabolites using the area under the peaks, the relative concentration of metabolites among control and drug treatment groups was determined as a mean and standard deviation (S.D.) (Additional file 2: Table S1) and log2-fold changes (Additional file 2: Table S2). The graphical output containing the heatmap correlation of significant metabolites which were associated with the drug response showed the direct and inverse correlations of different patterns of metabolites (Additional file 1: Figure S4a); the correlation and log2-fold change were used for creating the metabolic pathway as shown in Fig. S4b. Univariate analysis using *t*-test showed *p*-value of relative concentration of each metabolites in treatment group when compared with untreated control (Additional file 1: Figure S5) and some metabolites of important metabolic pathway were demonstrated (Additional file 2: Table S3).

**Fig. 6:**
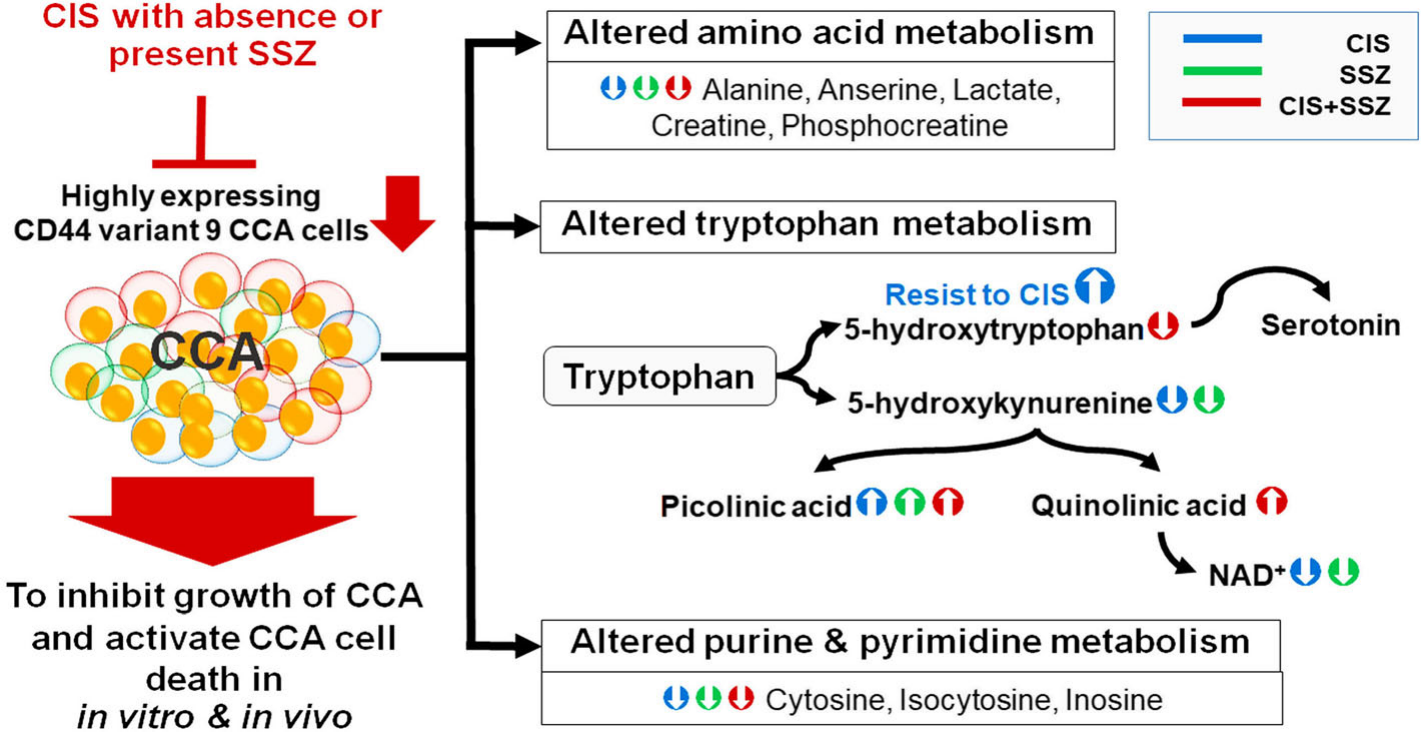
Proposed altered metabolic signature of drug treatment

Additionally, the CIS group was divided into 2 subgroups, including bad responders and good responders, depending on the drug response using tumor weight determination (median = 0.51 gram). The relative concentration of metabolites among CIS-treated nude mice in the bad responders (*n* = 2) and good responders (*n* = 2) showed a 5-hydroxytryptophan level (10658 and 5880, respectively, *p* = 0.556) and p-hydroxyphenylpyruvate (8079 and 3229, respectively, *p* = 0.338). There was a trend to increased values in bad responders. Conversely, the level of cis-aconitate (24,0473 and 26,8296, respectively, *p* = 0.889) had a trend to decreased values in bad responders. Although the metabolites of the bad responders and good responders showed no significant differences, the level of their metabolites after CIS combined with SSZ treatment was significantly different when compared with CIS treatment in bad responders (5-hydroxytryptophan: 820 and 10,658, *p* = 0.001; p-hydroxyphenylpyruvate: 1146 and 8079, *p* = 0.033; cis-aconitate: 55,9689 and 24,0473, *p* = 0.043). Therefore, the metabolic profiling alteration during different drug treatments presented a unique pattern between each group: i.e. control vs CIS, control vs SSZ, and control vs CIS plus SSZ. All pairs had a different pattern of metabolites associate with drug response. Moreover, metabolic signatures in the bad responder group had a different pattern to the good responders so that SSZ combined with CIS might modify the metabolic signatures and contribute to CCA suppression (Fig. 6).

## Discussion

Cholangiocarcinoma, especially in Ov-associated patients, has a high rate of recurrence and no effective chemotherapy [35, 36]; however, a recent report revealed a high level of CD44v9-positive cells in CCA patients [15]. Consequently, a combination of drugs might be used to prevent CCA recurrence. We report xCT-targeted CD44v9-CSCs therapy using SSZ (SSZ) in combination with CIS or GEM in CCA therapy. Our findings suggest that the combination of SSZ and CIS is more effective than CIS alone in both in vitro and in vivo models in Ov-associated CCA cell lines, with a reduction in CD44v9 expression. Our previous report revealed a negative association of CD44v9 and oxidative stress indicator (phospho-p38MAPK) in CCA patients related to Ov infection (76%) that was higher than CCA patients with no Ov association (52%). This evidence suggested that the redox regulation of Ov-associated CCA was mainly mediated via CD44v9-xCT system, but it was not found in non-Ov-associated CCA [15]. At present, there is no report for SSZ treatment in non-Ov-associated CCA. However, SSZ improved ROS-mediated apoptosis in CIS treatment in hepatocellular carcinoma (HCC) with high CD44v and xCT expression [37]. Similarly, a study of colorectal cancer indicated that SSZ could sensitize cancer cells to chemotherapeutic drugs [38]. In addition, 5-FU resistance was seen in the upregulation of CD44v9 expression by increasing intracellular glutathione and suppressing the drug-induced production of reactive oxygen species (ROS), and SSZ enhanced the drug sensitivity of CD44v9-expressing cells in gastric cancer [39]. Interestingly, the ratio of CD44v9 to CK-19 (proliferative marker) in a combination of CIS and SSZ was significantly less than CIS alone. These findings indicated that the proliferation of CD44v9-positive cells was reduced after CIS and SSZ treatment. Likely, SSZ would be effective to kill cancer stem-like CCA cells which are CD44v9-positive. Previously, the study of Seishima et al demonstrated that long-term sulfasalazine administration reduced proliferative CD44v9+ cells and increased the degree of differentiation of adenocarcinomas [19]. Recently, Shitara and coworkers showed a reduction of the levels of CD44v-positive cancer stem-like cells and GSH was observed, consistent with the mode of action of SSZ in CSCs [40]. Moreover, SSZ in xCT overexpressing non-small-cell lung cancer cells decreased cell proliferation and invasion in vitro and in vivo, and regulated metabolic requirements, especially glutamine metabolism [41].

A unique pattern of the metabolic profile in tumor tissues was seen in each treatment when compared with the untreated control. Interestingly, we found an increase in quinolinate for SSZ in the presence of CIS. Quinolinate or quinolinic acid is formed from tryptophan in the liver and the brain by the kynurenine pathway. The kynurenine pathway is involved in many diseases and disorders, including Alzheimer’s disease, amyotrophic lateral sclerosis, Huntington’s disease, AIDS dementia complex, malaria, cancer, depression, and schizophrenia, where imbalances in tryptophan and kynurenines have been found [42]. Similar to our results, a previous report revealed that SSZ inhibited NFκB-dependent upregulation of kynurenine pathway activity in the human placenta [43].

This study also demonstrated that untreated tumors had upper metabolite levels for the kynurenine pathway, including tryptophan, 3-hydroxykynurenine, and other products of the pathway such as picolinate. Picolinic acid or picolinate is a monocarboxylic acid that is an endogenous neuroprotectant and a natural iron and zinc chelator [44]. Picolinate or picolinic acid is one of the alternate end products of the kynurenine pathway, resulting from the enzymatic conversion of 2-amino-3-carboxymuconate semialdehyde by the enzyme 2-amino-3-carboxymuconate semialdehyde decarboxylase [45]. It can block quinolinic acid to induce neurotoxicity, but not the neuroexcitatory component [46**,** 47**]**. Compared with kynurenic acid, picolinic acid is less potent and appears to act via a different mechanism, attenuating calcium-dependent glutamate release and/or chelating endogenous zinc [48**–**50**]**.

These metabolites were increased in untreated cells. As a result, the imbalance in the pathway might be important in driving the progression of CCA. Outstandingly, quinolinate can be changed to NAD and continuously to NAD^+^, which is catabolized by quinolinic acid phosphoribosyltransferase (QPRT). We found quinolinate only in the SSZ plus CIS treatment. On the other hand, for CIS alone we did not see any separation peak of quinolinic acid within the control or CIS groups. We did, however, find that NAD^+^ was higher in the control when compared with CIS treatment. The prevention of apoptosis in human malignant glioma cells involves the QPRT enzyme via utilizing quinolinic acid for NAD^+^ synthesis. In addition, an upregulation of QPRT was associated with a poor prognosis in recurrence patients and resistance to oxidative stress induced by radiochemotherapy [51**]**. Quinolinate can enhance reactive oxygen species (ROS) formation in the tumor microenvironment by several mechanisms, including the formation of redox-active complexes with Fe^2+^ leading to lipid peroxidation [52]. Furthermore, the expression of most kynurenine enzymes is altered in breast cancer patients and the inhibitors of these related enzymes could be used as drugs in addition to the standard chemotherapy regimens, thus presenting a viable therapeutic approach [45**].** A previous study showed that alkylating agents or direct NAD+ synthesis inhibitors can deplete the level of intracellular NAD^+^, and QPRT is used as a potential therapeutic target in malignant gliomas [51**]**. These supporting studies indicate that SSZ can modify the kynurenine pathway via suppressing the synthesis of NAD^+^, consequently leading to improvement in CCA therapy with CIS.

Tryptophan is an essential amino acid which is mainly a part of two metabolic pathways: serotonin metabolism and kynurenine metabolism [53]. Tryptophan degradation occurs *via* the kynurenine pathway where two different enzymes, indoleamine-2,3-dioxygenase (IDO) and tryptophan-2,3-dioxygenase (TDO), catalyze the conversion of tryptophan into kynurenine, while tryptophan hydroxylase-1 (TPH-1) converts tryptophan to 5-hydroxytryptophan and provides precursors for serotonin biosynthesis [54, 55]. Our study demonstrated that hydroxytryptophan and hydroxyphenylpyruvate levels of CIS plus SSZ were reduced when compared with bad responders of CIS treatment, while the cis-aconitate level was increased. Hydroxytryptophan is the intermediate metabolite in the synthesis of serotonin, which mainly functions as a neurotransmitter to modulate neural signaling in a wide range of neuropsychological activities and is involved in cancer progression. Although it was previously reported that serotonin metabolism is dysregulated in cholangiocarcinoma progression and inhibition of serotonin synthesis could suppress the growth rate of non-Ov-associated cell line in an in vivo model [56, 57], blood plasma of intrahepatic CCA patients in the UK revealed the key metabolites involved in CCA progression that were orotate and orotidine in the pyrimidine pathway [58]. Moreover, it has been reported that indoleamine 2,3-dioxygenase (IDO), an important enzyme of the kynurenine pathway and functions to catabolize tryptophan to kynurenine, is induced by inflammation [59]. There are several evidences supporting the carcinogenesis mechanism of CCA with or without Ov was different. The data shows the upregulated genes of xenobiotic metabolism and chronic inflammatory responses were seen in Ov-associated CCA patients, including cytokine signaling, whereas non-Ov-associated CCA patients have upregulated expression of growth factor signaling, such as HER2 [7]. Taken together, the role of the kynurenine pathway might be different between CCA with or without Ov infection.”

Hydroxyphenylpyruvate is an intermediate in the metabolism of the amino acid phenylalanine and tyrosine and has a high level in primary epithelial ovarian cancer and metastatic tumors resulting from primary ovarian cancer [60]. Cis-aconitate is an intermediate in the tricarboxylic acid cycle (TCA) metabolism and the alteration of the tricarboxylic acid cycle (TCA) metabolism has been reported in many cancers including colorectal cancer [61] and lung cancer [62].

## Conclusion

In summary, we found that SSZ could improve CCA therapy by increasing cell sensitivity to CIS. This occurs by killing CD44v9-positive cells both in vitro and in vivo. Modification of the metabolic pathway was changed after SSZ treatment in the presence of CIS, mainly via the kynurenine pathway and purine and pyrimidine metabolisms. Importantly, the metabolic signatures of bad responders were altered after CIS combined with SSZ.

## Supplementary Information

**Additional file 1: Supplementary Figures**.

**Additional file 2: Supplementary Tables***.*

## Data Availability

The datasets generated during and/or analyzed during the current study are available from the corresponding author on reasonable request.

## Abbreviations

CCA: Cholangiocarcinoma
SSZ: Sulfasalazine
CIS: Cisplatin
GEM: Gemcitabine
Ov: *Opisthorchis viverrini*
CD44: Cluster of differentiation 44
CD44v9: CD44 variant 9
CSCs: Cancer stem-like cells
ROS: Reactive oxygen species
GSH: Glutathione
NMR: Nuclear magnetic resonance
STOCSY: Statistical total correlation spectroscopy
