# Supplementary material for: Sulfasalazine modifies metabolic profiles and enhances cisplatin chemosensitivity on cholangiocarcinoma cells in in vitro and in vivo models
Source: Cancer Metab. 2021 Mar 16;9:11. doi: 10.1186/s40170-021-00249-6 (PMC7968252; doi:10.1186/s40170-021-00249-6)
Supplement: Supplementary file 1 — Additional file 1: Supplementary Figures. [file 40170_2021_249_MOESM1_ESM.docx]

**Supplementary Figures**

**
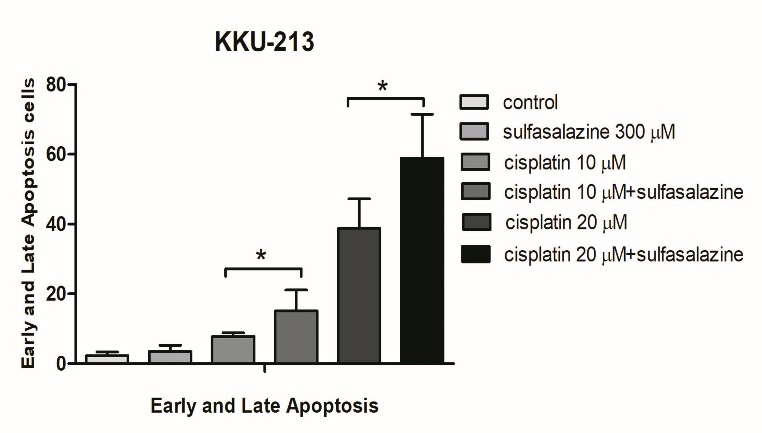
**

**Fig S1.** Cisplatin combined with sulfasalazine activates more CCA cell apoptosis than a single

treatment. The number of KKU-213 apoptotic cells with annexin-5 positive staining in early and

late stage counted and summarized as the mean±standard deviation of independent, triplicate

experiments.

**
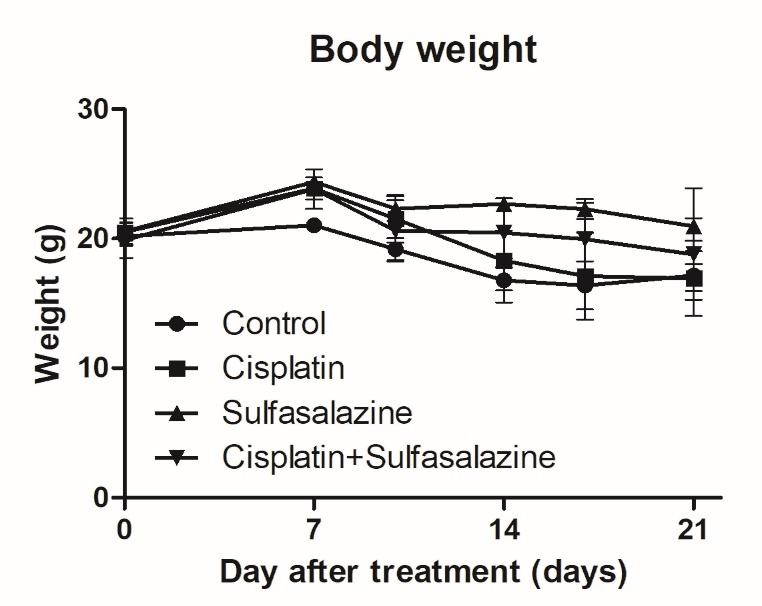
**

**Fig S2.** Body weight of nude mice between control and treatment groups

**
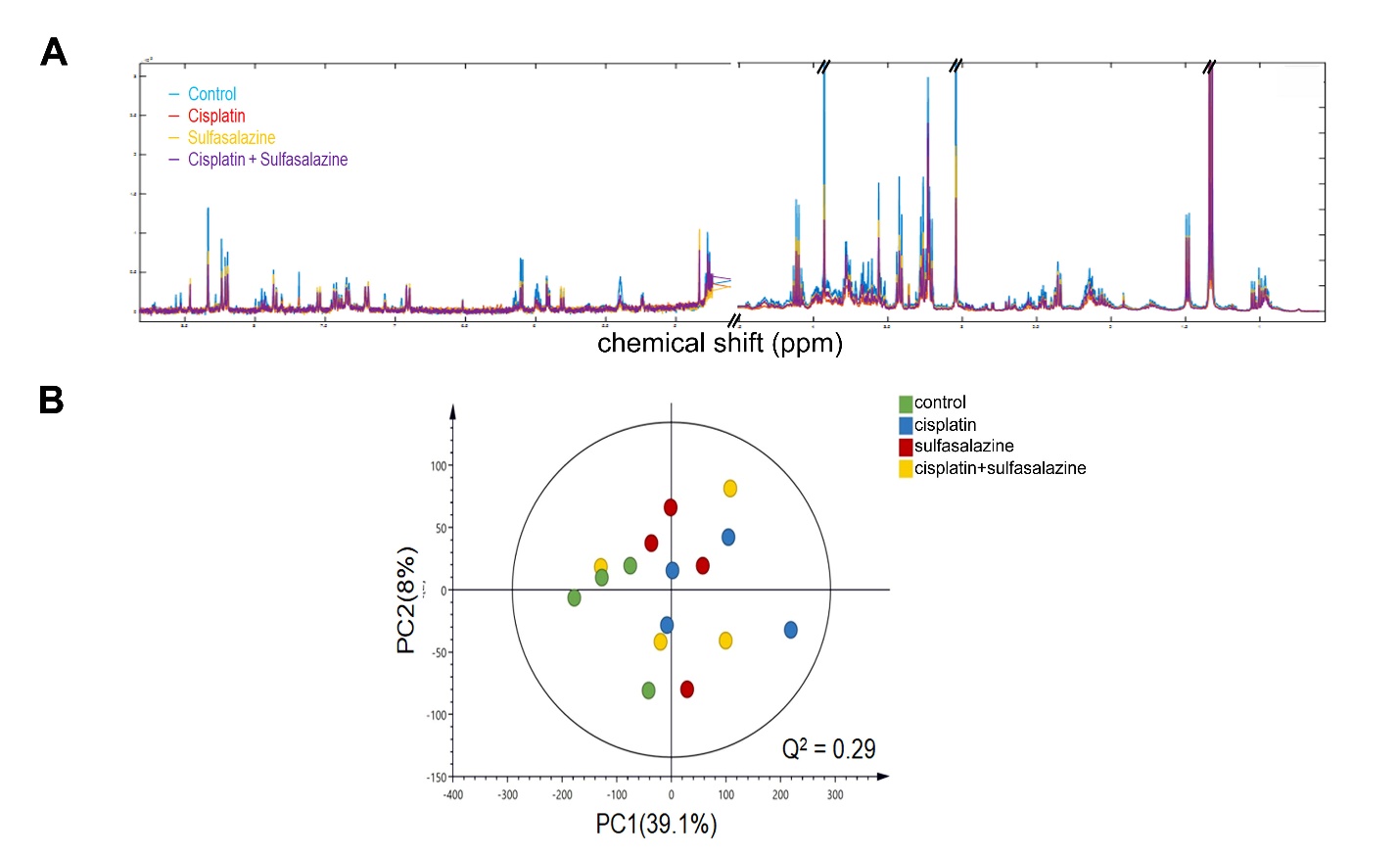
**

**Fig S3.** Difference metabolite profile between control and treatment groups. a) 1 H NMR spectra

plot shows median intensity of control and treatment groups, b) Principle Component Analysis

(PCA) plot of all groups shows the components of control did not differ when compared with

treatment samples.


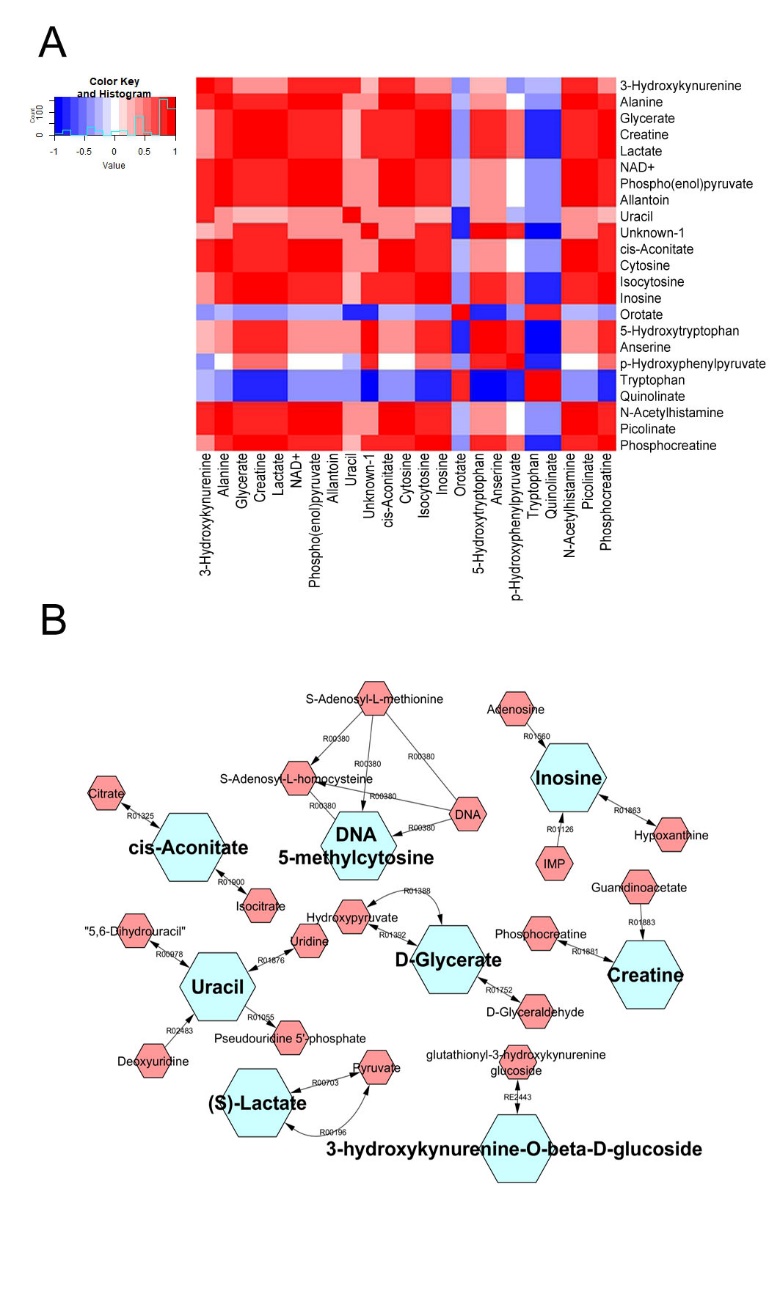


**Fig S4.** Network analysis demonstrates the association of significant metabolites. **a**) a heat-map

correlation of significant metabolites in all treatment groups compared with untreated group, b).

The metabolic network generated by MetScape and the proposed altered pathways in drug

response.


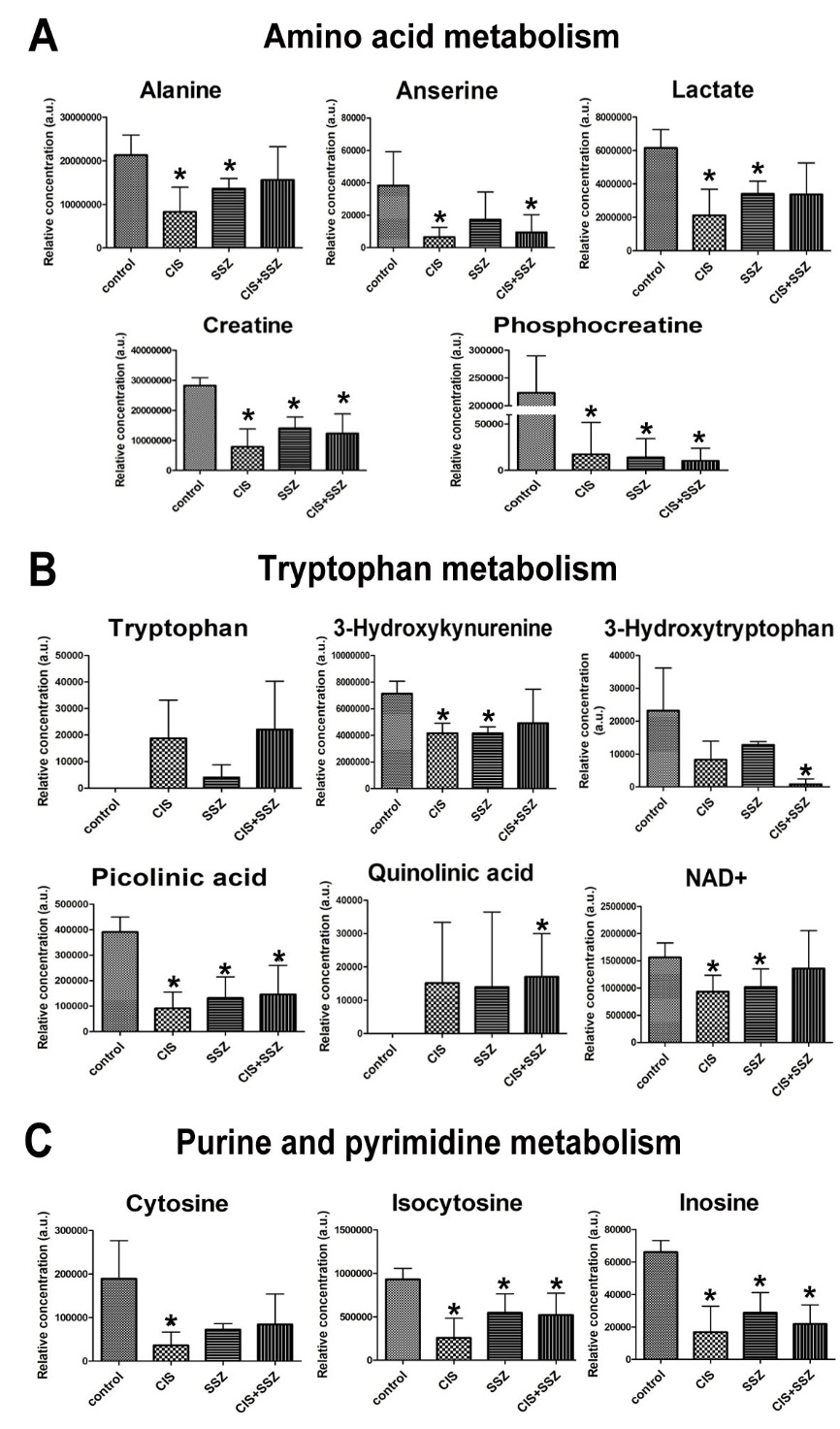


**Fig. S5.** Significant metabolic pathway of tumor tissue extraction between control and

treatment with cisplatin, or sulfasalazine, or a combination of both. The bar graphs present the

relative concentration of metabolites in a) amino acid metabolism, b) tryptophan metabolism and

c) the purine and pyrimidine pathway.
