# Supplementary material for: Sulfasalazine modifies metabolic profiles and enhances cisplatin chemosensitivity on cholangiocarcinoma cells in in vitro and in vivo models
Source: Cancer Metab. 2021 Mar 16;9:11. doi: 10.1186/s40170-021-00249-6 (PMC7968252; doi:10.1186/s40170-021-00249-6)
Supplement: Supplementary file 2 — Additional file 2: Supplementary Tables. [file 40170_2021_249_MOESM2_ESM.docx]

**Supplementary Tables**

**Table S1. Mean of metabolites in tumor tissue extraction**

|  | **control** | | **cisplatin** | | **sulfasalazine** | | **cisplatin+sulfasalazine** | |
| --- | --- | --- | --- | --- | --- | --- | --- | --- |
| **Metabolites** | mean | SD | mean | SD | mean | SD | mean | SD |
| 3-Hydroxykynurenine | 7134210 | 793393 | 4164187 | 638974 | 4162260 | 413282 | 4907453 | 2209264 |
| Alanine | 21292385 | 3949837 | 8243154 | 4906677 | 13602028 | 2009943 | 15591066 | 6607503 |
| Glycerate | 4278763 | 310605 | 2069600 | 1229294 | 2809584 | 409712 | 2767410 | 971724 |
| Creatine | 28207958 | 2278537 | 7843519 | 5160359 | 14094201 | 3174473 | 12336553 | 5598309 |
| lactate | 6155000 | 956203 | 2122300 | 1353935 | 3412720 | 651040 | 3368729 | 1640692 |
| NAD+ | 1562457 | 229251 | 932057 | 261542 | 1017510 | 288136 | 1357551 | 602666 |
| Phospho(enol)pyruvate | 68053 | 15312 | 19439 | 9554 | 20352 | 15076 | 27651 | 12214 |
| Allantoin | 388084 | 98210 | 119750 | 72632 | 137987 | 96799 | 139374 | 54258 |
| Uracil | 549403 | 246811 | 475083 | 137494 | 435955 | 206107 | 444493 | 234317 |
| Unknown-1 | 28794 | 7826 | 20983 | 13728 | 23094 | 23816 | 10791 | 15876 |
| cis-Aconitate | 658698 | 150620 | 254385 | 124967 | 524080 | 111381 | 559689 | 170240 |
| Cytosine | 189447 | 75410 | 36456 | 26222 | 72595 | 11845 | 84326 | 60457 |
| Isocytosine | 930400 | 109777 | 257439 | 195040 | 546815 | 188125 | 520151 | 217883 |
| Inosine | 66128 | 6020 | 16803 | 13809 | 28783 | 10696 | 21862 | 10117 |
| Orotate | 0 | 8297 | 5531 | 3686 | 8766 | 2744 | 16691 | 10038 |
| 5-Hydroxytryptophan | 23180 | 11270 | 8269 | 4891 | 12783 | 859 | 0 | 0 |
| Anserine | 38383 | 17954 | 2992 | 10589 | 17087 | 15068 | 293 | 18815 |
| p-Hydroxyphenylpyruvate | 7532 | 2254 | 5654 | 3285 | 8858 | 3444 | 0 | 0 |
| Tryptophan | 0 | 0 | 17527 | 14387 | 0 | 0 | 21769 | 16203 |
| Quinolinate | 0 | 0 | 0 | 0 | 0 | 0 | 22569 | 8204 |
| N-Acetylhistamine | 424809 | 142570 | 96839 | 47650 | 159913 | 37709 | 187111 | 113300 |
| Picolinate | 391011 | 51137 | 91544 | 55527 | 132475 | 71731 | 146131 | 98270 |
| Phosphocreatine | 223252 | 57603 | 0 | 0 | 493 | 27856 | 0 | 0 |

**Table S2. Fold change of metabolites in treatment groups compared with control**

| **Metabolite list** | **cisplatin** | **sulfasalazine** | **cisplatin+sulfasalazine** |
| --- | --- | --- | --- |
| 3-Hydroxykynurenine | -0.78 | -0.78 | -0.54 |
| Alanine | -1.37 | -0.65 | -0.45 |
| Glycerate | -1.05 | -0.61 | -0.63 |
| Creatine | -1.85 | -1.00 | -1.19 |
| lactate | -1.54 | -0.85 | -0.87 |
| NAD+ | -0.75 | -0.62 | -0.20 |
| Phospho(enol)pyruvate | -1.81 | -1.74 | -1.30 |
| Allantoin | -1.70 | -1.49 | -1.48 |
| Uracil | -0.21 | -0.33 | -0.31 |
| Unknown-1 | -0.46 | -0.32 | -1.42 |
| cis-Aconitate | -1.37 | -0.33 | -0.23 |
| Cytosine | -2.38 | -1.38 | -1.17 |
| Isocytosine | -1.85 | -0.77 | -0.84 |
| Inosine | -1.98 | -1.20 | -1.60 |
| Orotate | NA | NA | NA |
| 5-Hydroxytryptophan | -1.49 | -0.86 | NA |
| Anserine | -3.68 | -1.17 | -7.04 |
| p-Hydroxyphenylpyruvate | -0.41 | 0.23 | NA |
| Tryptophan | NA | NA | NA |
| Quinolinate | NA | NA | NA |
| N-Acetylhistamine | -2.13 | -1.41 | -1.18 |
| Picolinate | -2.09 | -1.56 | -1.42 |
| Phosphocreatine | NA | -8.82 | NA |

NA; No applicable.

**Table S3. Univariate analysis of the relative concentration of metabolites in the treatment groups compared with the control**

| **Metabolite list** | **cisplatin** | **sulfasalazine** | **cisplatin+sulfasalazine** |
| --- | --- | --- | --- |
| 3-Hydroxykynurenine | 0.003** | 0.003** | 0.180 |
| Alanine | 0.012* | 0.035* | 0.257 |
| Glycerate | 0.049* | 0.003** | 0.069 |
| Creatine | 0.003** | 0.001** | 0.011* |
| lactate | 0.007** | 0.008** | 0.054 |
| NAD+ | 0.021* | 0.045* | 0.612 |
| Phospho(enol)pyruvate | 0.005** | 0.009** | 0.013* |
| Allantoin | 0.010* | 0.020* | 0.014* |
| Uracil | 0.669 | 0.564 | 0.613 |
| Unknown-1 | 0.433 | 0.716 | 0.147 |
| cis-Aconitate | 0.012* | 0.263 | 0.480 |
| Cytosine | 0.033* | 0.073 | 0.111 |
| Isocytosine | 0.004** | 0.030* | 0.038* |
| Inosine | 0.004** | 0.004** | 0.001** |
| Orotate | 0.099 | 0.052 | 0.026* |
| 5-Hydroxytryptophan | 0.102 | 0.208 | 0.014* |
| Anserine | 0.033* | 0.168 | 0.044* |
| p-Hydroxyphenylpyruvate | 0.449 | 0.600 | 0.040* |
| Tryptophan | 0.080 | 0.590 | 0.069 |
| Quinolinate | 0.045* | 0.154 | 0.012* |
| N-Acetylhistamine | 0.023* | 0.044* | 0.067 |
| Picolinate | 0.000** | 0.003** | 0.015* |
| Phosphocreatine | 0.002** | 0.003** | 0.004** |

*p-value<0.05, **p-value<0.01
